# Supplementary material for: Effect of IGFBP-4 during In Vitro Maturation on Developmental Competence of Bovine Cumulus Oocyte Complexes
Source: Animals (Basel). 2024 Feb 21;14(5):673. doi: 10.3390/ani14050673 (PMC10931042; doi:10.3390/ani14050673)
Supplement: Supplementary file 1 [file animals-14-00673-s001.zip › animals-2844951-supplementary.pdf]

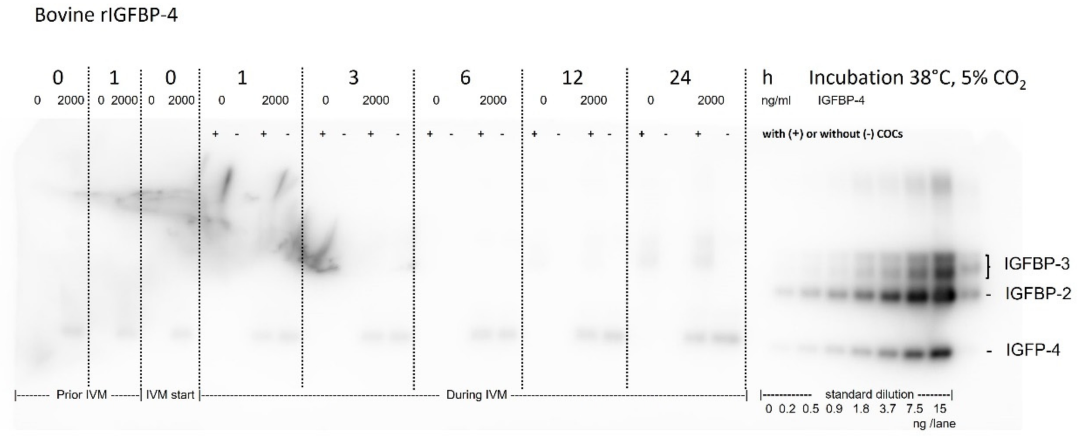

Figure S1. Supplementary figure. (A) Analysis of in vitro maturation (IVM) medium with and without addition of recombinant bovine insulin-like growth factor protein 4 (rbIGFBP-4). rbIGFBP-4 can be detected as a band in all samples in which rbIGFBP-4 (2,000 ng/ml) was added, prior to the start of IVM, samples were taken; (0) directly after IVM medium preparation, (1) one hour after incubation. Prior IVM start (0) a sample was taken before cumulus-oocyte complexes (COCs) were placed in the maturation plates. During IVM; samples were taken from wells in the presence (+) or absence (-) of COCs at one (1), three (3), six (6), twelve (12) and twenty-four (24) h after IVM started. The western ligand blot image was cropped at the level corresponding to IGFBP-4, an original image of the gel it is provided in the supplementary file (Figure 6). (B) analysis of intact of recombinant human IGFBP-4 (rhIGFBP-4), rbIGFBP-4, and recombinant human (IGFBP-2). (C) Protein quantity determination of rh- and rbIGFBP-4 and rhIGFBP-2.
